# Rare Species Are Valued Big Time

**DOI:** 10.1371/journal.pone.0005215

**Published:** 2009-04-22

**Authors:** Elena Angulo, Franck Courchamp

**Affiliations:** Ecologie, Systématique et Evolution, UMR CNRS 8079, Université Paris Sud, Orsay, France; University of Pretoria, South Africa

## Abstract

**Background:**

It has recently been postulated that the value humans place on rarity could cause the extinction of rare species. This is because people are willing to pay the high costs of exploiting the last individuals. Many hobbies, such as ecotourism or the keeping of exotic pets may cause this effect – known as the anthropogenic Allee effect. However, the entire theory relies on the insofar undemonstrated assumption that people do value rarity.

**Methodology/Principal Findings:**

In order to quantify how much people valued rare species relative to common ones, we created online slideshows of photographs of either rare or common species on an Internet web site. The slideshow with photographs of rare species attracted more visitors, and visitors spent, in general, more time waiting to view it.

**Conclusions/Significance:**

We provide evidence that people value rare more than common species. As we did not target consumers of a specific market, this finding suggests that the anthropogenic Allee effect is likely be driven by a large part of the population. Given the substantial participation in our online experiment, we highlight the potential of the world wide web resource as a tool for conservation action. However, the evidence presented here that the general public value rare species, combined with the assumption that anthropogenic Allee effect is operating, implies that conservationists should be prudent when using rarity to promote conservation.

## Introduction

The exploitation of rare and endangered species may result in their extinction, if people who greatly value rarity can drive an increase in the economic incentives to exploit the last individuals, thereby creating a positive feedback loop [1]. This recently described concept, known as the anthropogenic Allee effect, shows how humans attributing value to rarity could precipitate the extinction of rare species [2]. Historically, economic theory suggested that rare species would be safe from overexploitation, as the costs of exploiting rare species would prevent a viable economic market [3]. However, under the anthropogenic Allee effect theory, less abundant species could suffer disproportionately from exploitation if their rarity makes them systematically more valuable [2], [4].

Different activities may drive an anthropogenic Allee effect: collections and trophy hunting, in which the rarity of a species is directly related into an exponential increase in their value [5]–[7]; luxury items, traditional medicine and exotic pets, in which the perception of rarity increases the owners prestige and, in turn, increases people willingness to pay even high prices [8]–[10]; negative impacts of ecotourism on species via disturbance would be mostly focused on fashionable species, most of which correspond to already endangered species [2]. Such higher value of rare species remains, however, to be demonstrated.

The difficulty in demonstrating high value of rarity stems from three main points. The first is the metrics of the value itself. The most obvious choice is currency (e.g. euros), but several studies have shown that willingness to pay is not a satisfactory metric to assess the value people invest in goods [see for ex. 11]. The second difficulty is to identify a good for which a value can be attributed in the framework of an experimental design, and which can be compared for rare and common species, without endangering the species concerned. The third difficulty is the need to obtain unbiased and honest responses from a sufficiently high number of subjects, implying that they must not be aware that their choices are being monitored. These three main obstacles may explain why, despite its seemingly intuitive straightforwardness, the higher value attributed to rarity in living species has never been demonstrated.

Here, we performed an experiment to quantify the hypothesized higher value attributed to rare species compared to a common one (all other things being equal). We created online slideshows of photographs of either rare or common species on an Internet web site. We distinguished three different indications of value: attractiveness of each slideshow (measured by the percentage of visitors to each slideshow), perseverance to download it (measured by the number of attempts for each slideshow), and finally patience while waiting to download it (measured by the time spent for each slideshow). While visitor's attraction measures directly the value of a given species (rare or common), time spent and number of attempts are a way of estimating personal investment, which we assumed proportional to the value given to each species. Our results unambiguously confirm the added value of rarity and the likely generality of the concept of the anthropogenic Allee effect.

## Results

### Attractiveness of photographs of rare species

We provided online slideshows of photographs of either rare or common species. Visitors to the web site were given the choice between the two slideshows. Upon clicking the link to the selected slideshow, an upload progress bar opened. However, the slideshow never started, and the time passed from starting to cancel the download was automatically recorded. The program also recorded, for each attempt, the time and date, as well as the position (which changed randomly) of the selected slideshow on the webpage (rare or common). The IP number of the computer through which access was made was automatically coded and recorded so that we could differentiate attempts from individual computers ( = visitors, see methods). A total of 4967 different attempts were recorded in the two week duration experiment. Nine events were disregarded as the recording was erroneous due to an unusual system configuration. We also removed data for durations >20 hours and finally obtained a total of 4941 data that came from 2560 different visitors.

**Figure 1:**
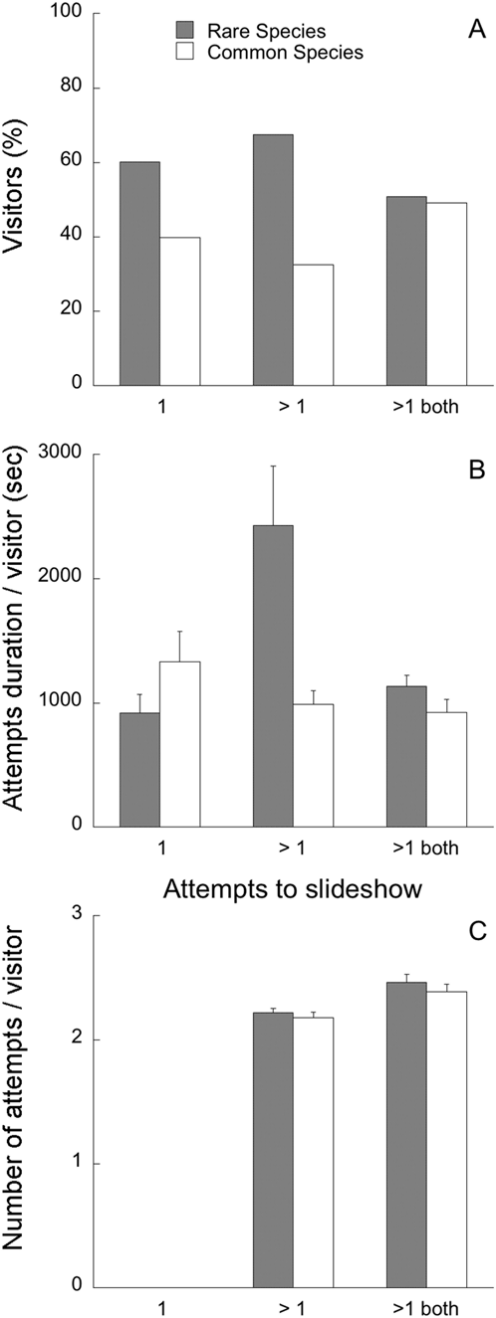
Behaviour of visitors having to choose between slideshows of rare or common species. We show (A) attractiveness, (B) patience and (C) perseverance of visitors. Data comes from visitors who attempted to open only one slideshow type, once (1) or multiple times (>1), or their first choice when they attempted to open both slideshows (>1 both). Error bars indicate standard errors.

Almost half (48.4%) of the 1240 visitors made only one download attempt. Of these, 60.2% made an attempt to see the rare species, while the rest tried the common species (ℵ^2^ = 4.13, p = 0.042, Fig. 1a). Within the other half of the visitors, 347 (13.5%) made several attempts to open only one slideshow, and 67.4% of them tried to open the rare species (ℵ^2^ = 12.17, p<0.001, Fig. 1a). The rest of the visitors (N = 973, 38.0%) made several attempts and tried both slideshows at least once. Among them, 50.9% tried the rare first (ℵ^2^ = 0.03, p = 0.862, Fig. 1a).

### Patience awaiting for the rare species photographs

Visitors remained between one second to more than 20 hours on the page with the progress bar. The time waited (in minutes) fit a gamma distribution with a peak in the first two minutes. The first 6 minutes (which corresponded to the time it took for the download bar to be entirely filled) accounted for 3112 data (63.0% of the total) and the first 4 hours accounted for 4561 data (92.3% of the total). It was obvious that the longest attempts were made by those of visitors who left the slideshow open in the background while not paying attention to it.

**Figure 2:**
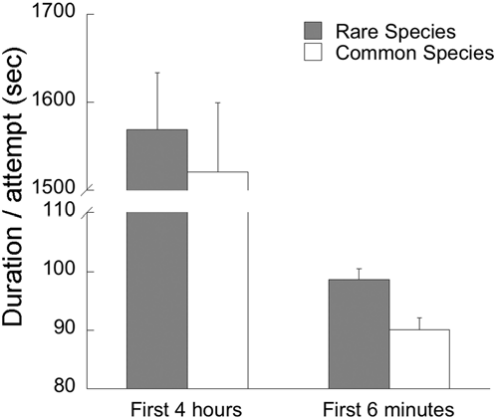
Effect of the slideshow type (rare or common species) on the time spent by visitors. We show data for attempts shorter than 6 minutes and for attempts between 6 minutes and 4 complete hours. Error bars indicate standard errors.

Firstly, we looked at all attempts by classifying them by their duration (shorter or longer than 6 minutes, the time upon completion of the progress bar). Results regarding the first 6 minutes showed that only the type of slideshow (rare or common) significantly affected the time spent downloading the slideshow (ℵ^2^ = 9.38, p = 0.002, N = 3068 attempts, Fig. 2); visitors spent more time waiting for the rare species slideshow to open. Results regarding visitors that cancelled the slideshow between 7 minutes and 4 complete hours showed no relationship with the type of slideshow (ℵ^2^ = 0.0, p = 0.982, N = 1802, Fig. 2), but visitor's age, sex and level of studies were significantly related to the time spent: men spent more time waiting for the slideshow to open than women (ℵ^2^ = 4.60, p = 0.032, N = 1802 attempts), as did visitors between 26–35 years old (ℵ^2^ = 12.28, p = 0.006; N = 1802 attempts) and people of higher level of studies (ℵ^2^ = 4.04, p = 0.044; N = 1802 attempts). These variables were recorded before accessing the slideshow webpage (see methods).

Secondly, we focussed on the total visit duration per visitor and divided visitors into those attempting to access either slideshow once, those attempting to access the same slideshow more than once and those attempting to access both slideshows more than once. Among the visitors that made only one attempt to the slideshow, the total time spent was higher for the common species (ℵ^2^ = 12.72; p<0.001; N = 1240, Fig. 1b). Among the visitors who attempted to access the same slideshow more than once, the total time spent was higher for the rare species (ℵ^2^ = 27.38; p<0.001; N = 347, Fig. 1b). Visitors that made more than one visit to the web page and attempted to see both slideshows, spent more time trying to access that of the rare species, although the trend was not significant (ℵ^2^ = 2.76, p = 0.097, N = 1944, Fig. 1c).

### Perseverance to see the rare species photographs

Among the visitors that made more than one attempt, 347 tried the same slideshow every time, and they did so between two and five times. The total number of attempts for each slideshow by visitors attempting to open the rare species slideshow was higher but not significantly different than for the visitors attempting to open the common species slideshow (ℵ^2^ = 0.56, p = 0.453, N = 347; Fig. 1c). The 973 visitors who tried to see both slideshow types several times, did so between two and 32 different times, with more attempts for the rare species, although not significantly (ℵ^2^ = 2.94, p = 0.087, N = 1944, Fig. 1c).

## Discussion

The experiments performed in this study aimed to validate a key assumption underpinning the concept of the anthropogenic Allee effect; that people value rarity of wildlife. Preference for a rare species could maintain a sufficient demand as to overcome the high exploitation cost for the last individuals, putting it into a vicious circle of overexploitation, and finally leading to its extinction [2]. Here, we have shown that more than half of the 2560 visitors would have preferred to see the slideshow with photographs of the rare species. Moreover, within the first six minutes waiting to download the slideshow (time the downloading bar took to fill up), people waited longer for the rare species slideshow compared to that of the common species. These results were not affected by their age, sex, or level of education.

Our experiment provided no details of the species supposedly displayed in the slideshows and was based on a comparison between rare or common species, thus rarity is clearly identified as the cause of the preference. This is unusual when searching for relationships between rarity and value (i.e. correlations). For example, it has been shown that caviar price in markets increased as sturgeon (*Acipenser baerii*) abundance decreased [12]; or fleet size engaged in whale watching increased as killer whale (*Orcinus orca*) abundance decreased [13]. In these two examples, as well as in the examples on other anthropogenic Allee effect activities [2], it is difficult to separate correlation from causation. Because our experiments were based on two slideshows for which exactly the same (or lack of) information was given, it is actually the comparison between the value of rare and common that we studied. In this regard, any potential bias should logically be similar for the two slideshows, leaving only the rarity attribute as the cause of potential differences. Also, the independence of our results from specific species confers higher generalization to our conclusions.

The value attributed by people to species is generally measured by the price people would, in theory, accept to pay (the willingness to pay). Such metrics have been criticized and the use of non-monetary criteria has been advocated [11], [14]. In this paper, we propose different metrics to assess the relative value of rarity: the (first or unique) choice of a slideshow to be viewed, the time spent in waiting for the slideshow to appear, and the number of attempts to open the slideshow. Two of these three variables were significantly related to rarity. The main goal of this article was to show that the general public gives more value to rare species relative to common ones. Our results demonstrate that visitors had an increased interest in rare species and we suggest that this interest is potentially linked to an anthropogenic Allee effect. Whether this increased interest could drive a higher economic value should be the focus of future studies.

Within visitors who made a unique choice, more than 60% tried the rare species slideshow, and within visitors who attempted several times to see the same slideshow, almost 70% selected the rare species one. Differences were less important for those who tried to see both slideshows several times; probably at some stage (more than five times), the type of species had become less important to the visitor than the success of seeing any slideshow at all. We assumed that the first choice is a direct indication of the people's value for a species.

Rarity also affected the time spent waiting for the slideshow to begin within the first 6 minutes. We assumed that time spent waiting is proportional to personal interest, so that here people were more interested in rarity. When we classified visitors by their number of visits to the slideshows, we obtained contrasting results for the time expended waiting for the slideshow. Visitors waited more for the rare slideshow when making more than one attempt, but waited more for the common slideshow when making only one attempt to download a slideshow. This last result could be explained if, when confronted with a choice of two items, people often “leave the best until last”. This has been observed in another study of rarity (over commonness) of wildlife based luxury goods [12]. When realizing that their second choice also would not download, those visitors would have given up more rapidly. Overall, and because visitors were not aware that their decisions were being monitored, we believe these parameters were not biased and reflected the real relative value visitors attributed to rarity.

We believe our online experiment was original because we were able to obtain a high sample of unbiased people. Assuming that each response from a given IP address came from a single visitor, we obtained a total of 2560 visitors. Even if a visitor could access the slideshow from different machines or different visitors could log on the same machine, these scenarios should represent a small percentage of the visits and their expected effect should not be very important given the very large sample size we obtained. We tried to diversify the likely recipients of the online slideshow experiment by contacting large newsgroups in random subjects, or by asking recipients to forward their message, but the bulk of the primary email list was the university of Paris XI staff and students. Our online questionnaire requested information on the visitor's education level; even if our final sample was biased in favour of higher education levels, this information was taken into account during the statistical analyses. In summary, we believe these results demonstrate the higher value attributed to rare species by the general public, as opposed to specific wildlife trade users who were not targeted here. Although it is quite likely that different cultural roots, political and/or social interests could also be biasing them [15], our results should hold for most industrialized countries, and probably beyond. It is also noteworthy that our original, web-based, approach generated a large amount of data: during two weeks and with no advertising other than targeted email contact, at least 2560 different visitors were interested to see online slideshows of photographs of rare/common species, enough to endure through the notoriously painful process of answering three questions before accessing the slideshow page. Given the significant participation in our online experiment, we highlight the potential of the world wide web as a tool for conservation actions.

Beside these methodological recommendations, one may extract two main findings from this study. The first one is that rarity by itself is an important trait for the general public when related to animal species, and this should continue to be used as a tool for the conservation of rare and endangered species. The second implication of this study, however, is that as rare species are more valued than common ones, there is a high likelihood of existence of an anthropogenic Allee effect [2] in diverse wildlife related human activities. The particular threat this effect poses on rare species is sufficiently disturbing for conservationists to use caution when disclosing rarity, as well as to begin a dialogue about the measures that can be adopted to protect rare species from this new threat.

## Materials and Methods

### Online slideshow experiment design

We created a web page (http://www.ese.u-psud.fr/diapos/) to which visitors were invited to view high quality images of rare and common animals. To access the slideshow pages, visitors had to first go through a page consisting of a very short questionnaire asking their sex, age (six categories: less than 15 years old; 15–25; 25–35; 35–50; 35–65 and more than 65 years old) and education level (four categories: no degree; general degree; bachelor degree and master degree). Upon reply, the slideshow page appeared offering the possibility to view two different slideshows. The only indication of the slideshows' contents was that one was showing rare species and the other common species. The slideshow links were just two similar buttons labeled with the words ‘rare’ or ‘common’. The two links were positioned to minimize bias in the first selected choice and the position of the two links (upper-left or lower-right) was randomly generated each time the page was loaded. Clicking on either of the slideshow links opened as small window with a cancel button in which a progression bar indicated the proportion of the slideshow that had been downloaded as well as a cancel button. The progression of the download was rapid until halfway so as to encourage visitors to stay, but then slowed exponentially. The bar was entirely filled after six minutes, but nothing happened (the slideshow still appeared to be downloading). The visitors could cancel the downloading at anytime, in which case they were redirected to an error page indicating that they had cancelled the downloading before completion, and they were given a link to the rare/common slideshow page to try again.

We recorded automatically the response of the short questionnaire together with the time and date, the slideshow position, the selected slideshow(s) and the duration from choice to cancellation for each attempt. We automatically coded the IP number so that we could differentiate attempts from each machine, which we supposed to represent a single visitor. After authorization by the ethical committee of the CNRS and the University Paris Sud, we sent emails to the students and staff of the university, as well as to many newsgroups of nature, sport or photography users, asking them to forward the message as much as possible. The test lasted for two weeks in March and April 2006. Upon completion, another email was sent to explain the experiment, with an invitation to view a real (this time) slideshow of more than 300 photos of animals (http://www.ese.u-psud.fr/epc/conservation/pages/explication.html).

### Attractiveness of each slideshow type

We measured the visitor's attractiveness to the rare slideshow, based on the proportion of visitors selecting the rare slideshow as their only choice or as their first choice. In the latter case, we distinguished visitors that attempted to view the same slideshow every time and the ones that tried both slideshows (in which case we considered the nature of their first attempt only). We compared statistically the percentages of visitors selecting the rare or the common slideshow by calculating a ℵ^2^ (between observed *vs.* expected values).

### Patience awaiting for each slideshow

We measured the patience of visitors, based on the relative duration (in time) before cancellation of each slideshow. We firstly focused on the duration before and after 6 minutes, the time the progress bar was fully filled (i.e. the download was supposed to be complete). We analysed this data using a generalized linear model with a gamma distribution and a log link function (GLM_G_) for the dependent variable (time). We included the visitor as a repeated measure, to handle the possible covariance structure given by multiple visits of the same visitor. We also included four more independent variables: the position of the rare species slideshow (right or left), and the sex, age and education level of the visitor. Before doing so, we re-grouped the six levels of the age categorical variable into four levels only, to homogenize the sample size: extreme data with the smallest sample sizes were grouped with their next level. Similarly we reduced to two the four categories of the education variable: we merged the two lower categories (no degree and general degree) and the two higher categories (bachelor and master degree). We performed a backward stepwise regression, but the main effect (rare or common) was always maintained even when not significant. In a first analysis, we took into account only visits that were cancelled before the bar was fully filled (within the first 6 minutes). In a second analysis, we considered only the attempts that were cancelled after that, up to four hours. We believe that including periods longer than 4 hours were not realistic measures of visitor persistence or interest in the slideshow.

Secondly, we analyzed the duration of all attempts for either slideshow for each visitor. To do this, we divided visitors that made only one attempt to either the rare or common slideshow, visitors that made more than one attempt to the same slideshow and visitors that made more than one attempt to both slideshows. In the first case, we performed a GLM_G_ with the time duration as the dependent variable and the type of slideshow as the independent variable. In the second case, we performed the same analysis but the dependent variable was the sum of the time duration of all attempts for each visitor. In the third case, we included the visitor as a repeated measure in the model, so that we compared time duration for each slideshow within visitors.

### Perseverance for each slideshow

We measured the perseverance of visitors, based on the total number of attempts by each visitor to open each slideshow. Similarly to the previous analyses, we firstly analyzed the visitors that made attempts at opening only one slideshow type (either the rare or the common). We performed two GLM_G_, using the total number of visits as dependent variables and the type of slideshow as the independent variable. This allowed us to compare number of attempts between visitors that only attempted to view the rare or the common slideshow. We secondly analyzed the cases when a visitor visited both slideshows. We performed the same analysis but the visitor was introduced in the model as a repeated measure, so that we compared these values within visitors.

Computations were performed with STATISTICA 6.0 [16] and the SAS package (GENMOD, v. 9.1.3., [17]).
